# Sociodemographic, personality and symptomatologic profiles associated with an increased likelihood of suicidal risk in patients hospitalized for recurrent depressive disorders

**DOI:** 10.1192/j.eurpsy.2021.465

**Published:** 2021-08-13

**Authors:** R. Kalinovic, G. Vlad, O. Neda-Stepan, M. Dinescu, C. Giurgi-Oncu, I. Enatescu, V.R. Enatescu

**Affiliations:** 1 Biochemistry, “Victor Babes” University of Medicine and Pharmacy, Timisoara, Romania; 2 Psychiatry I, “Pius Brinzeu” Emergency County Hospital-Psychiatric Clinic, Timisoara, Romania; 3 Psychiatry, “Victor Babes” University of Medicine and Pharmacy, Timisoara, Romania; 4 Neonatology And Childcare, “Victor Babes” University of Medicine and Pharmacy, Timisoara, Romania

**Keywords:** depression, suicidal risk, sociodemographic

## Abstract

**Introduction:**

According to WHO statistics, 800,000 suicides occur annually, representing the second leading cause of death in people aged 15 to 29. The contributing factors for suicidal risk are multifactorial and multileveled.

**Objectives:**

We aimed to analyze the predictive value of distinct sociodemographic, personality and symptomatology characteristics in predicting the presence of suicidal risk in patients hospitalized for the analyzed mood disorder.

**Methods:**

A longitudinal retrospective case-control study was performed on medical data records of 90 patients admitted in the Timisoara Psychiatric Clinic during 2018 – 2020. Besides the parametric and non-parametric statistical analyses, logistic binary regression analyses were done.

**Results:**

Patients with suicide risk tended to be younger (p = 0.039), without intimate partnership (p < 0.001), current smoker (p = 0.038) and to present psychotic symptoms at some moments during the psychiatric disorder. 51 (56.7%) of the total patients have presented different degrees of suicidal risk (from suicidal ideation to suicide attempt). Patients with suicide risk tended to be younger (p = 0.039), without intimate partnership (p < 0.001), current smoker (p = 0.038) and to present psychotic symptoms at some moments during the psychiatric disorder. Personality traits has not influenced suicidal risk. Presence of intimate partner (OR = 0.135; p < 0.001) and the presence of psychotic symptoms during recurrent depression (OR = 7.309; p = 0.004) have presented predictive value on suicide risk.

**Conclusions:**

Psychiatrist practitioners should be aware of the clinical and sociodemographic characteristics that put recurrent depressive patients at risk of suicidal behaviors.

**Disclosure:**

No significant relationships.

